# Attitudes toward learning communication skills among Iranian medical students

**DOI:** 10.1186/s12909-024-05140-8

**Published:** 2024-02-25

**Authors:** Naser Yousefzadeh Kandevani, Ali Labaf, Azim Mirzazadeh, Pegah Salimi Pormehr

**Affiliations:** 1 Yas Hospital Complex, Tehran University of Medical Sciences, Tehran, Iran; 2 Present Address: Imam Khomeini Hospital Complex, Tehran University of Medical Sciences, Tehran, Iran; 3 Department of Medicine, Department of Medical Education, School of Medicine, Tehran University of Medical Sciences, Tehran, Iran

**Keywords:** Communication skills, Medical students, Attitude

## Abstract

**Background:**

Attitudes determine behavior, and alterations in attitude may result in behavioral changes. Medical students would benefit from learning communication skills. This study aimed to determine the attitude of medical students toward the importance of learning communication skills and the training courses and their role in contributing.

**Methods:**

In this cross-sectional study, 442 medical students from three different levels of medical training were enrolled. Students in the first 4 years of the medical program were classified as basic sciences and physiopathology students, those in the fifth and sixth years were classified as clerkship students, and those in the last three terms of medical training were classified as interns. The attitude among these three groups was assessed by the Communication Skills Attitude Scale (CSAS) questionnaire, and the contributing factors were determined.

**Results:**

The mean total points for attitude in positive and negative aspects were 50.7 and 30.9, respectively showing a positive attitude toward communication skills among medical students. The median scores of the scales Important in Medical Content, Excuse, Learning, and Overconfidence varied significantly from highest to lowest, respectively. Gender, educational level, ethnic origin, language, family burden, paternal literacy, history of presence in communication skills courses, self-report from communication skills, and need to further learning in this era showed significant association with attitude (*P* < 0.05).

**Conclusions:**

It may be concluded that generally, medical students have a positive attitude toward communication skills, and this perspective is a multi-factorial entity that programming according to the various related factors would help to attainment of additional communication capabilities among medical students.

## Background

Attitude is the key precursor of behavior, and attitude alterations may result in behavioral changes. A brief educational program in communication skills would be beneficial for medical students to develop better relationships and may improve some other aspects of communication skills in health care professionals [1–4]. Regarding the importance of capabilities in physicians to develop communication with patients, this relationship has been centered on attention for responsible subjects and health sector managers [5–7]. This ability has been part of the educational curriculum to release trained physicians in this era for interpersonal and physician-patient relationships [2, 3, 8]. It has been altered from an earlier compact one-week pattern to a longitudinal design across different courses during the general practitioner training phase [4, 5, 8]. The main question in this era is the role of this training to develop better practice among undergraduate medical students and general physicians for communication skills with patients and colleagues. Assessment of attitudes about the importance of communication skills and attitude among medical students about the educational courses would indirectly demonstrate the applicability of such learning programs [8]. It would also help in the development of a better curriculum. For this reason, attitude assessment is crucial to determine how medical students think about the importance of the issue and if these training courses are beneficial and should be continued.

In this study, we investigate the attitudes of medical students at Tehran University of Medical Sciences (TUMS) towards communication skills, recognizing the pivotal role of these skills in shaping effective patient-physician interactions. The evolution of educational programs in communication skills underscores their increasing importance, prompting questions about their impact on undergraduate medical students and general physicians. Our research aims to fill this gap by evaluating how medical students perceive the importance of communication skills, the effectiveness of training courses, and the potential influence of demographic factors on these attitudes. Through a comprehensive assessment, we seek to indirectly illuminate the applicability of communication skills learning programs and contribute insights for ongoing curriculum development. This study responds to the need for a deeper understanding of medical students’ attitudes, offering valuable insights to inform future curriculum development and contribute to the discourse on optimizing communication skills training in medical education.

## Methods

### Study design and population

In this cross-sectional study, 470 medical students in three groups from different levels of medical students were enrolled, including students in the first 4 years as basic sciences and physiopathology levels, those in fifth and sixth years of medical course as clerkship level, and those in last three medical training terms or undergraduate medical students.

### Study tool

The attitude among these three groups was assessed by Communication Skills Attitude Scale [CSAS] questionnaire that was initially introduced by Rees et al. [6] and previously validated among Iranian subjects [9] after permission from Rees et al. It includes 26 questions, including 13 negative and 13 positive attitude items with 5-scale Likert pattern resulting in total points from 13 to 65 in each section. The higher score is positive, and the lower point in negative numbers demonstrated a more positive attitude. Questions number 2, 3, 6, 8, 11, 13, 15, 17, 19, 20, 22, 24, and 26 were related to negative attitude and the numbers 1, 4, 5, 7, 9, 19, 12, 14, 16, 18, 21, 23, and 25 were related to positive attitude. Four dimensions of important in medical context (questions number 1, 4, 5, 9, 10, 14, 16, 19, 21, 23, 25), excuse [2, 6, 8, 10–12], learning [7, 13–16], and overconfidence [3, 17, 18] were considered in this study [9].

### Sample size

There are approximately 9000 medical students in Iran. To be 95% confident that the true value of the estimate will be within 5 percentage points of 0.5 the required sample size is 369. In order to achieve the stated level of accuracy, this number of actual responses must be obtained. This means 369 or more medical students are needed to have a confidence level of 95% that the real value is within ±5% of the measured/surveyed value. For calculating this sample size the following formula was used:

$$\text{n}=\frac{z^2\times\widehat{\text{p}}\left(1-\widehat{\text{p}}\right)}{\widehat\varepsilon}$$

$${\textrm{n}}^{\prime }=\frac{\textrm{n}}{1+\frac{{\textrm{z}}^2\times \hat{\textrm{p}}\left(1-\hat{\textrm{p}}\right)}{{\hat{\varepsilon}}^2\textrm{N}}}$$

Where,

z is the z score (1.96).

ε is the margin of error (0.5).

N is the population size (9000).

p̂ is the population proportion.

### Study definitions

Attitude is defined as a constellation of emotions, beliefs, and behaviors directed towards a specific object, individual, entity, or occurrence. Rooted in experiences or upbringing, attitudes wield a potent influence on behavior, shaping individuals’ actions across diverse situational contexts [19].

Communication skills denote an individual’s capacity to precisely convey information to another individual or group. These aptitudes play a pivotal role in problem-solving, fostering positive relationships, and facilitating effective therapeutic interactions. Encompassing verbal and non-verbal communication modalities, active listening, empathetic engagement, and the adeptness to tailor language to suit specific audiences, communication skills constitute an indispensable facet for sustaining healthy interpersonal connections. Moreover, they are actively sought by employers across diverse professional domains [20].

### Study variables

Demographic and background variables were also added to the questionnaire, including age, gender, marital status, educational level, total educational average, another academic or vocational history, job, parental literacy level, ethnicity, religion, family burden, type of high school, and history of training in communication skills courses. Also, the self-view about communication skills and the need for improvement in these capabilities was questioned. The association between demographic variables with four dimensions was assessed, including besides the positive and negative attitude responses. After a brief explanation of the project and its purposes, questionnaires were distributed after each level’s theoretical classes. Students were asked to fill them out and return them before leaving the class. A written consent form was attached to the questionnaire as the first page, and it was emphasized that participation in the study was optional.

### Statistical analysis

Descriptive statistics were presented by mean ± standard deviation (SD) for continuous variables and frequency (percentage) for categorical variables. Scale scores were compared using the violin plot and notch plot. The bar plot was used to compare the total score of scales by demographic variables including birthday, sex, marital status, ethnicity, language, and religion. The t-test was used to compare the mean of scales between different levels of demographic variables and shown with asterisks. The analysis of variance (ANOVA) was used to compare the total score of scales across factors. The multiple ANOVA was used to evaluate the adjusted impact of variables on the total score of scales. All analyses were performed using R (version 4.1.2) and SPSS (version 25). *P*-values less than 0.05 were regarded as statistically significant.

## Results

A total of 442 cases (237 females) were included in the study. Of these, 387 (87.6%) were single. The ethnicity of 336 (76.0%) study participants was Fars, and the language of 342 (77.4%) was Persian. 419 (94.8%) cases were Muslim. There were 57 participants who had experience in the job before enrolling in the study. Thirty (6.8%) participants were the only children in their household. Other descriptive statistics were presented in Table 1.

**Table 1:** The descriptive statistics of demographic variables and characteristics factors

	Frequency (%)
Total cases	442
Birth year	
1991–1997	139 (31.4)
1997–2003	297 (67.2)
Sex	
Male	204 (46.2)
Female	237 (53.6)
Marital status	
Single	387 (87.6)
Married	52 (11.8)
Ethnicity	
Fars	336 (76.0)
Others	103 (23.3)
Language	
Persian	342 (77.4)
Others	73 (16.5)
Religion	
Islam	419 (94.8)
Others	22 (5.0)
Job experience	
No	380 (86.2)
Yes	57 (12.9)
Family burden	
Single	30 (6.8)
More children	408 (92.3)
University mark	
< 14	13 (2.9)
14–16	292 (66.1)
≥ 17	127 (28.7)
High school field	
Mathematics	71 (16.1)
Experimental science	371 (83.9)
Pre university field	
Mathematics	15 (3.4)
Experimental science	426 (96.4)
High school type	
Federal	46 (10.4)
Others	393 (88.9)
Pre university type	
Federal	43 (9.7)
Others	396 (89.6)
Father’s job	
Doctor	68 (15.4)
Medical staff	11 (2.5)
Other	354 (80.1)
Mother’s job	
Doctor	37 (8.4)
Medical staff	17 (3.8)
Other	357 (80.8)
Paternal literacy	
Middle school	24 (5.4)
High school	95 (21.5)
Academic	321 (72.6)
Maternal literacy	
Middle school	51 (11.5)
High school	124 (28.1)
Academic	261 (59.0)
Presence in CS courses	
No	316 (71.5)
Yes	116 (26.2)
Self-report from CS	
Excellent	34 (7.7)
Good	198 (44.8)
Medium	168 (38.0)
Poor	36 (98.6)
Need for further learning	
No	58 (13.1)
Yes	377 (85.3)

**Fig. 1:**
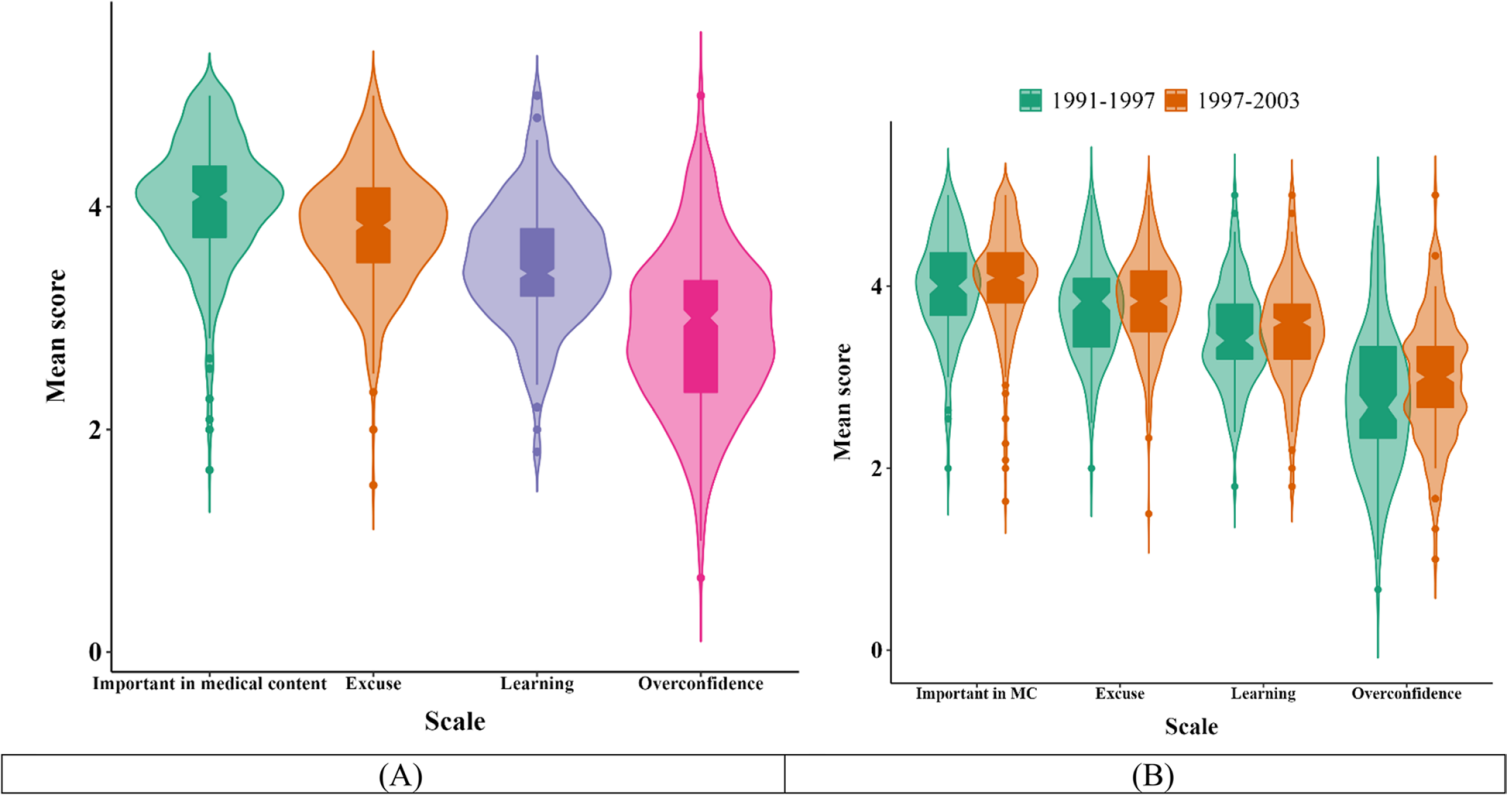
**A** The total mean score of scales, (**B**) the mean score of scales by years

In the next step, the total and mean score of scales were presented using violon plot in Fig. 1. The highest mean scores were associated with scales including Important in medical context, Excuse, Learning, and Overconfidence. Regarding the notch plot, the mean score of participants born between 1997 and 2003 was higher than those born between 1991 and 1997.

**Fig. 2:**
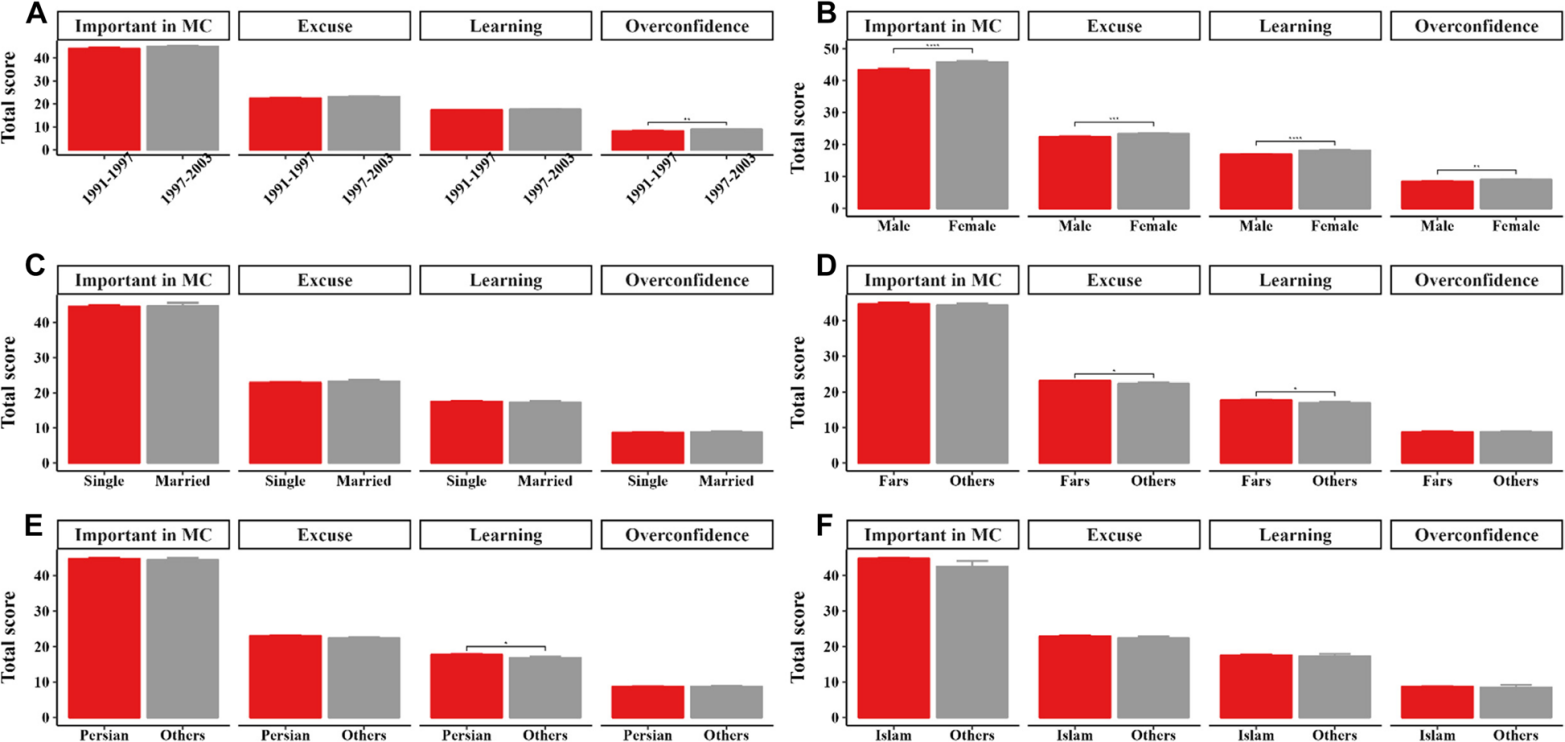
The total score of scales by **A**) birth year, **B**) gender, **C**) marital status, **D**) ethnicity, and **E**) language, and **F**) Religion

As shown in Fig. 2, the total score of Overconfidence was significantly higher for participants born between 1997 and 2003. Compared to men, higher total score of scales was observed for women. The total score of Excuse and Learning were higher in Fars ethnicity compared to other ethnicities. Persian participants had higher Learning scores.

Table 2 showed the mean and standard deviation of total scores for scales related to different levels of variables. There were significant relationships between Important in medical content and variables including family burden (*p* < 0.001), maternal literacy (*p* = 0.014), self-reported CS (*p* < 0.001), and need for further learning (*p* < 0.001). The relation between Excuse scale and variables family burden (*p* = 0.017), presence in CS (*p* = 0.027) and need for further learning (*p* < 0.001) were significant. Significant associations were observed between Learning scale and family burden (*p* = 0.047), presence in CS (*p* = 0.010), and need for further learning (*p* < 0.001). Overconfidence scale was only related with presence in CS (*p* = 0.040).

**Table 2:** Comparing the total score of scale between different levels of variables

	Important in MC		Excuse		Learning		Overconfidence	
	Mean ± SD	*P*-value	Mean ± SD	*P*-value	Mean ± SD	*P*-value	Mean ± SD	*P*-value
Total	44.63 ± 6.11		22.87 ± 3.10		17.51 ± 2.80		8.68 ± 2.15	
Job experience		0.802		0.698		0.349		0.668
No	44.79 ± 5.84		22.86 ± 3.05		17.60 ± 2.72		8.73 ± 2.11	
Yes	44.58 ± 6.89		23.04 ± 3.54		17.23 ± 3.08		8.60 ± 2.25	
Family burden		**< 0.001**		**0.017**		**0.047**		0.280
Single	40.63 ± 8.29		21.57 ± 4.21		16.53 ± 3.10		9.10 ± 1.84	
More children	44.95 ± 5.84		22.97 ± 3.00		17.59 ± 2.78		8.66 ± 2.16	
University mark		0.876		0.937		0.475		0.914
< 14	44.00 ± 10.65		22.62 ± 3.33		16.62 ± 3.78		8.46 ± 2.07	
14–16	44.56 ± 5.99		22.91 ± 3.02		17.53 ± 2.76		8.68 ± 2.17	
> 17	44.80 ± 5.76		22.86 ± 3.21		17.61 ± 2.83		8.72 ± 2.12	
High school field		0.885		0.269		0.907		0.265
Mathematics	44.54 ± 7.15		22.49 ± 3.70		17.55 ± 2.39		8.42 ± 2.30	
Experimental science	44.65 ± 5.90		22.94 ± 2.98		17.51 ± 2.88		8.73 ± 2.12	
Pre university field		0.362		0.869		0.439		0.683
Mathematics	43.20 ± 7.42		22.73 ± 2.96		18.07 ± 2.19		8.47 ± 1.92	
Experimental science	44.66 ± 6.06		22.87 ± 3.11		17.50 ± 2.83		8.70 ± 2.16	
High school type		0.304		0.157		0.579		0.731
Federal	45.54 ± 5.09		23.48 ± 2.43		17.74 ± 2.19		8.59 ± 2.25	
Others	44.56 ± 6.22		22.79 ± 3.17		17.50 ± 2.87		8.70 ± 2.14	
Pre university type		0.925		0.991		0.671		0.470
Federal	44.58 ± 5.12		22.86 ± 2.98		17.35 ± 2.22		8.47 ± 2.25	
Others	44.67 ± 6.22		22.87 ± 3.12		17.54 ± 2.86		8.71 ± 2.14	
Father’s job		0.247		0.813		0.761		0.565
Doctor	44.57 ± 6.93		22.97 ± 3.17		17.66 ± 3.05		8.57 ± 2.06	
Medical staff	44.91 ± 4.41		23.45 ± 2.77		18.09 ± 2.39		8.09 ± 2.43	
Other	44.69 ± 5.95		22.87 ± 3.10		17.52 ± 2.76		8.72 ± 2.15	
Mother’s job		0.108		0.234		0.745		0.758
Doctor	46.14 ± 7.00		23.49 ± 3.36		17.92 ± 3.43		8.89 ± 2.01	
Medical staff	43.41 ± 6.85		21.94 ± 3.56		17.59 ± 2.00		8.47 ± 2.10	
Other	44.66 ± 5.97		22.92 ± 3.06		17.54 ± 2.80		8.67 ± 2.11	
Paternal literacy		0.736		0.111		0.356		0.726
Middle school	43.42 ± 5.91		22.17 ± 2.91		17.13 ± 2.64		8.33 ± 2.06	
High school	45.74 ± 5.58		23.40 ± 3.09		17.85 ± 2.71		8.69 ± 2.25	
Academic	44.41 ± 6.26		22.76 ± 3.12		17.44 ± 2.85		8.69 ± 2.13	
Maternal literacy		**0.014**		0.458		0.620		0.237
Middle school	44.45 ± 5.75		22.59 ± 2.82		17.27 ± 3.12		8.41 ± 2.04	
High school	45.00 ± 5.54		23.17 ± 2.93		17.43 ± 2.68		8.50 ± 2.22	
Academic	44.50 ± 6.48		22.84 ± 3.21		17.64 ± 2.81		8.83 ± 2.14	
Presence in CS courses		0.106		**0.027**		**0.010**		**0.040**
No	44.22 ± 6.28		22.82 ± 3.07		17.32 ± 2.79		8.64 ± 2.13	
Yes	46.14 ± 5.21		23.12 ± 3.15		18.23 ± 2.68		8.77 ± 2.18	
Self-report from CS		**< 0.001**		0.231		0.294		0.708
Excellent	43.59 ± 7.99		21.85 ± 4.13		16.71 ± 3.43		8.29 ± 2.48	
Good	45.30 ± 5.72		23.02 ± 3.00		17.70 ± 2.70		8.76 ± 2.07	
Medium	44.50 ± 5.76		22.93 ± 2.79		17.46 ± 2.80		8.68 ± 2.04	
Poor	42.94 ± 7.63		22.67 ± 3.83		17.58 ± 2.77		8.64 ± 2.79	
Need for further learning		**< 0.001**		**< 0.001**		**< 0.001**		0.601
No	41.81 ± 8.26		20.95 ± 3.98		16.10 ± 3.08		8.55 ± 2.56	
Yes	45.19 ± 5.50		23.19 ± 2.82		17.77 ± 2.66		8.71 ± 2.09	

A t-test was used to compare the total score of binary variables. Analysis of variance was used to compare the total scores of scales between variables with more than two variables

*MC* Medical content

Table 3 showed the adjusted impact of factors on the total score of scales. Accordingly, the adjusted impact of gender, ethnicity, family burden, high school type, pre university type, presence in CS courses, self-reported from CS, and need for further learning on the Important in medical content were significant. Gender, family burden, high school type, pre university type, and presence in CS course had significant effect on the Excuse. The adjusted effect of gender, ethnicity, and need for further learning on the Learning scale was significant.

**Table 3:** The multiple analysis of variance for evaluating the impact of factors on the total score of scales

	Important in MC		Excuse		Learning		Overconfidence	
	Β	*P*-value	β	*P*-value	β	*P*-value	β	*P*-value
Gender (female vs. male)	2.51	< 0.001	0.92	0.003	1.31	< 0.001	0.41	0.075
Ethnicity (others vs. Fars)			−0.61	0.092	−0.81	0.017		
Family burden (More children vs. single)	4.84	< 0.001	1.44	0.018	0.84	0.135		
High school field (experimental Sciences vs. mathematics)	−1.42	0.105					0.45	0.150
Pre university field (experimental Sciences vs. mathematics)	3.23	0.055						
High school Type (others vs. federal)	−3.64	0.019	−1.82	0.021				
Pre university Type (others vs. federal)	4.32	0.008	2.07	0.012				
Presence in communication skills courses (yes vs. no)	1.96	0.004	2.12	< 0.001	0.50	0.123		
Self-report from communication skills (good vs excellent)	−1.25	0.314						
Self-report from communication skills (medium vs excellent)	−2.55	0.047						
Self-report from communication skills (poor vs excellent)	−3.70	0.017						
Need to further learning (yes vs. no)	4.33	< 0.001			1.37	0.001		
Mother’s education (high school vs. middle school)							0.39	0.299
Mother’s education (academic vs. middle school)							0.71	0.052

Variables were selected by using the Generalized Akaike Information Criterion (AIC) in stepwise method

## Discussion

Developing communication competencies with patients and coworkers is necessary for today’s physicians [13]. We believe this qualification distinguishes successful doctors from others. Although a physician’s personality and state of being and mental health could substantially affect this skill, is it still a skill that could have been tough for medical students in medical schools [14]. The first steps for getting students ready to become good communicators are knowledge and principles of practical interpersonal communication skills and extending these skills in communication with patients and coworkers [21]. Still, attitudes toward using these skills in daily practice are undeniably crucial. Forming attitudes towards the importance of learning and using communications skills is a multifactorial entity. Background cultures and demographic data are the associated factors that might influence attitudes [10]. Although we cannot change anything in this matter, information on these measurable associations can allow us to adjust or reform curriculums to accommodate the needs of students.

We used the CSAS questionnaire to assess students’ attitudes. C. Rees and her colleagues in 2002 designed this tool, and we have validated its Persian version [9]. Information on the background and cultural context that students come from was asked to investigate those factors’ influence on attitudes. Four domains of the Persian version of CSAS were importance, excuse, learning, and overconfidence considered in assessing attitudes. This widespread application of the questionnaire within [9, 15] and beyond the borders of Iran [9, 11, 12, 16–18, 22–27] attests to its cross-cultural and international validity. The instrument’s versatility in capturing attitudes toward communication skills suggests its effectiveness in transcending geographical and cultural boundaries.

Female students had a more positive attitude in all four aspects. This matter was also reported in previous studies [10, 28, 29]. We believe female doctors pay more attention to the fillings of their patients, so they have a more positive attitude towards the importance of communication skills.

Age and, consequently, the educational level of students did not significantly influence their attitude in all domains we measured. Other studies differ in their result on this issue. K.B Wright and colleagues reported that first-year medical students have the same attitudes towards the importance of learning communication skills as fourth-year students [29, 30]. Some studies have been shown that older students at a higher level of study have a more positive attitude [31]. Conversely, it has been shown that Younger students had a more positive attitude [6]. These differences might be due to contextual variations of each country and even differences in the journey medical students walk through in different universities and hospitals.

Our findings suggest an intriguing trend among students who are only children, as evidenced by significantly lower scores on questions related to excuse and learning regarding the importance of CS in medicine. While our initial interpretation points towards the potential impact of an isolated upbringing, we acknowledge the need for a more thorough exploration and alignment with existing literature to substantiate this observation. The notion that being a single child might influence attitudes toward CS in medicine deserves further scrutiny to identify and understand potential confounding factors that may contribute to this observed trend. A comprehensive review of relevant literature will be undertaken to strengthen the validity of this observation and provide a more nuanced interpretation of the results.

A significant difference of attitudes towards communication skills is present in students who participated in a communication skills course at university. These courses seem to make a good impression and cause more positive attitudes towards CS. This might be the impact of training to develop more positive perspectives. Other studies have also reported the critical role of the previous training course [29, 32, 33]. However, only 26.6% of medical students said a presence in such classes. Still, regarding the mandatory status of this course across the medical education period, it may be presumed that students with a negative report about the presence in such classes have forgotten their presence in these courses. Hence, those who had remembered their present have more positive attitudes naturally. This matter develops some uncertainties about the positive role of participation in short and cross-sectional classes.

In multivariable analysis, Female students, Fars’s ethnicity, having siblings, Students of federal high schools, the ones presented in CS courses, and finally, students who believed their CS need further improvement to have significantly more positive attitudes towards the importance of learning communication skills.

Discussion on how and why demographic differences or different personality of students might affect their attitudes and performance on using communication skills is essential and valuable. On the other hand, we suggest that social determinants like society’s overall conditions and some known factors like social capital, the respect, social and financial support that students get from the community, and role models with good communication skills are among the most critical contributors in forming attitudes. Although making a difference or controlling these factors are somehow beyond our abilities as medical teachers and program directors. The impact of social determinants on students’ attitudes and filling about humanitarian skills are complicated. Students might not be aware of the indirect influence of social factors. These entities need in-depth investigations, although intervention on these issues is not straightforward.

## Conclusions

In conclusion, our study of 442 medical students illuminates the intricate dynamics of attitudes toward communication skills, revealing a predominantly positive outlook, particularly among female students and those of Fars ethnicity. The figures, including violin plots and notch plots, effectively visualize the distribution and significance of various factors influencing attitudes. Notably, students who were only children displayed lower scores on aspects related to excuse and learning, prompting a call for further exploration into the impact of an isolated upbringing. Multivariable analysis underscored the significant influence of demographic factors, such as ethnicity and educational background, while highlighting the lasting impact of communication skills courses on shaping positive attitudes. The observed trends emphasize the importance of self-awareness in attitude formation, as students acknowledging a need for further improvement in communication skills expressed heightened positivity. As we consider the implications of our findings, recognizing the complexity of social determinants, our study lays the groundwork for future interventional trials aimed at understanding and altering communication skills perspectives to enhance competencies among medical students. Furthermore, our results carry broader societal implications, offering insights that can inform tailored interventions in medical education, ultimately contributing to the enhancement of effective communication in healthcare settings, improved patient-physician interactions, and the overall advancement of public health and well-being.

## Data Availability

The datasets analysed during the current study are available from the corresponding author upon reasonable request.

## Abbreviations

CSAS: Communication Skills Attitude Scale
TUMS: Tehran University of Medical Sciences
